# Supplementary material for: Causal Relationship between Aging and Anorexia Nervosa: A White-Matter-Microstructure-Mediated Mendelian Randomization Analysis
Source: Biomedicines. 2024 Aug 16;12(8):1874. doi: 10.3390/biomedicines12081874 (PMC11351342; doi:10.3390/biomedicines12081874)
Supplement: Supplementary file 1 [file biomedicines-12-01874-s001.zip › Supplementary Figure/Figure S8/Leaveoneout plot of MR analysis for TL to ubm-b-1711.pdf]

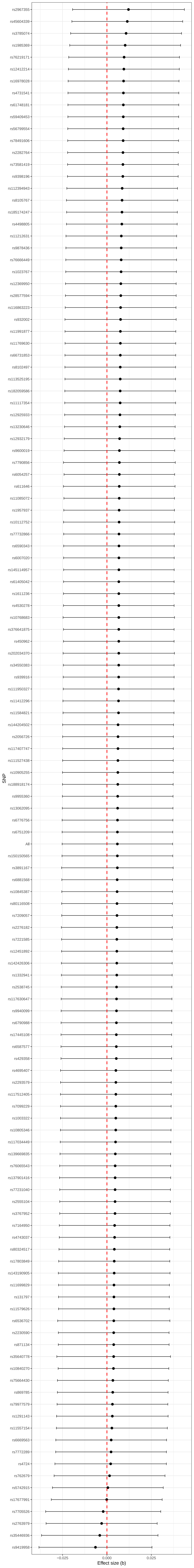

rs2967355
rs45604339
rs3785074
rs1985369
rs76219171
rs12412214
rs16978028
rs4731541
rs61748181
rs59409453
rs56799554
rs78491606
rs2282764
rs73581419
rs9398196
rs112394943
rs8105767
rs185174247
rs4498805
rs11212631
rs9878436
rs76666449
rs1023767
rs12369950
rs28577594
rs116863223
rs932002
rs11991877
rs117769630
rs66731853
rs8102497
rs113525195
rs182059586
rs111117354
rs12925933
rs13230646
rs12932179
rs9600019
rs7790856
rs6054257
rs611646
rs11085072
rs1957937
rs10112752
rs77732866
rs6590343
rs6007020
rs145114957
rs61405042
rs1611236
rs4530278
rs10768683
rs376641875
rs450962
rs202034370
rs34550383
rs939916
rs111950327
rs11412296
rs11584821
rs144204502
rs2056726
rs117407747
rs111527438
rs10905255
rs188918174
rs9955360
rs13062095
rs6776756
rs6751209
All
rs150150565
rs3891167
rs6881568
rs10845387
rs80116508
rs7209057
rs2276182
rs7221585
rs12451892
rs142426306
rs1332941
rs2538745
rs117630647
rs9940099
rs6790988
rs17445108
rs6587577
rs429358
rs4695407
rs2293579
rs117512405
rs7099229
rs1003322
rs10805346
rs117034449
rs139669835
rs76065543
rs137901416
rs77231040
rs2555104
rs3767952
rs7164950
rs4743037
rs80324517
rs17803849
rs143190905
rs11699829
rs131797
rs11579626
rs6536702
rs2230590
rs871134
rs35640778
rs10840270
rs75664430
rs869785
rs79977579
rs1291143
rs11557154
rs6669563
rs7772289
rs4724
rs762679
rs57429915
rs17677991
rs7705526
rs2763979
rs35446936
rs9419958
SNP
-0.025
0.000
0.025
Effect size (b)
